# Direct Healthcare Cost of Ischemic Stroke Management in Morocco

**DOI:** 10.7759/cureus.42180

**Published:** 2023-07-20

**Authors:** Mohammed Omari, Moncef Maiouak, Nassiba Bahra, Ibtissam El Harch, Mohammed Youbi, Latifa Belakhel, Loubna Abousselham, Nada Otmani, Belahcen Mohammed Faouzi, Nabil Tachfouti

**Affiliations:** 1 Laboratory of Epidemiology, Clinical Research and Community Health, Faculty of Medicine and Pharmacy, Sidi Mohamed Ben Abdellah University, Fez, MAR; 2 Department of Epidemiology and Disease Control, Ministry of Health and Social Protection, Rabat, MAR; 3 Department of Neurology, Hassan II University Hospital, Fez, MAR

**Keywords:** morocco, direct medical cost, neurology, ischemic stroke, cost-of-illness

## Abstract

Background: Strokes are a group of heterogeneous conditions that can cause lasting brain damage, long-term disability, or even death. In Morocco, the management of this disease generates important expenses and increases the financial burden on health care. In order to rationalize the expenses and to direct the budgetary policy in healthcare, we aimed to estimate the cost of ischemic stroke (IS) management in Morocco through this study.

Methods: A cost-of-illness study was conducted between March 2018 and March 2019 at the neurology department of the Hassan II University Hospital, Fez. We included all patients who were admitted, during this period, to the department for IS. The collected data included sociodemographic information, and all details regarding the patient’s medical management (diagnosis, treatment, etc.). The cost was estimated using a "bottom-up micro-costing" approach with a societal perspective.

Results: A total of 267 individuals were included in this study with a female predominance (56.6%); the mean age was 66.93 ± 14.83 years. The total cost of ischemic stroke management per patient per year was estimated at $3674.32 ± 1340.81, with a high share related to hospitalization at $1415.06 ± 1015.53. A statistically significant association was found between total cost and age (p=0.014), National Institutes of Health Stroke Scale (NIHSS) score (p≤0.001), and length of hospitalization (p≤0.001); however, no association was found with other factors (sex, complication, Rankin score, etc.).

Conclusion: Ischemic strokes are relatively frequent in Morocco. Their management generates an important cost, which is influenced by several factors such as severity of the disease and the duration of hospitalization. This cost can be decreased by rationalizing the expenses and acting on various risk factors of ischemic strokes.

## Introduction

Strokes are a group of heterogeneous medical conditions classified into three categories: cerebral infarctions (80%-90% of strokes), cerebral hemorrhages (10%-20%), and subarachnoid and meningeal hemorrhages (representing less than 2% of all strokes) [1]. Around the world, the annual incidence of stroke is approximately 16 million new cases, responsible for 5.7 million deaths [2]. Stroke is the second leading cause of death in the world (fourth in industrialized countries), the second leading cause of dementia (after Alzheimer's) and the leading cause of acquired motor disability in adults [3].

In Arab countries, the annual incidence of stroke ranges from 27.5 to 63 per 100,000 people and the prevalence ranges from 42 to 68 per 100,000 people, with the case fatality rate at 30 days ranging from 10% to 17.5%. The most common risk factors reported are hypertension, diabetes mellitus, hyperlipidemia, and heart disease [4]. The regional age-standardized point prevalence and death rates of stroke in the Middle East and North Africa (MENA) are 1537.5 (95% CI 1421.9-1659.9) and 87.7 (95% CI 78.2-97.6), respectively, per 100,000 people; the regional age-standardized disability-adjusted life year (DALY) rate in 2019 was 1826.2 (95% CI 1635.3-2026.2) per 100,000 people. High systolic blood pressure, high body mass index, and ambient particulate air pollution are the three biggest contributors to the burden of stroke in the MENA region [5].

In Morocco, an epidemiological survey carried out in two Moroccan metropolitan cities (Casablanca and Rabat) showed that the crude prevalence of stroke was 284 per 100,000 people, and ischemic stroke (IS) accounted for 70.9% of all types of stroke [6]. A recent systematic review of stroke epidemiological and etiological profiles showed that the most frequent etiologies were atherosclerosis and cardioembolic disease; the mortality rate varied in the acute phase from 3% to 13%, and the three-month mortality ranged from 4.3% to 32.5% [7]. Over the last decade, considerable progress has been achieved in diagnostic methods and therapeutic measures designed to reduce the impact of acute IS, particularly with the development of revascularization techniques [8].

The cost of stroke management varies; in low- to middle-income countries, the average direct medical cost is estimated to range from US$416 in Senegal [9] to US$4860 in Nigeria [10]. In Cameroon, this cost was estimated at €1451 for strokes in general and €1073 for ischemic strokes [11], while in Netherlands, this cost was as high as US$6845 [12].

Disease burden studies could help policymakers understand the economic costs of a particular disease. Hence, disease burden studies have an essential role to play in public health to formulate and prioritize healthcare policies and allocate healthcare resources by estimating the total costs that can be incurred by the diseases [13]. Considering the scarcity of healthcare resources, cost-of-illness (COI) studies on stroke care are needed to provide insights into the distribution of the cost and its impact on the national healthcare expenditure. Because investigations on the economic impact of stroke are lacking in Morocco, this study aimed to estimate its direct medical cost and identify important variables influencing the cost in a Moroccan region.

## Materials and methods

Study design and population

A cross-sectional COI study was conducted among patients admitted for stroke or transient ischemic attacks in the neurology department of the Hassan II University Hospital of Fez, Morocco, in 2019. The study included both new and old cases of both types of stroke, referred or admitted, followed up for more than 12 months. A prevalence-based approach was adopted to estimate the cost of illness. This approach estimates costs occurring concurrently with overall prevalent cases over a specified time period, usually a year. The prevalence-based approach is useful when the study’s purpose is to draw attention of the health policy planners or decision-makers to burden conditions [14,15].

Data collection

Data were collected by resident physicians from the electronic record integrated into the hospital information system, using a pre-established questionnaire. It included (1) sociodemographic data and medical and surgical history, (2) stroke clinical characteristics and (3) healthcare resource utilization during 12 months following diagnosis, including hospitalization, consultation, tests performed, medical treatment, dosages, commercial names and duration of medication, as well as medical procedures. We also conducted a short discussion with a vascular neurologist to ensure the accuracy of items and costs, and to collect data not available in medical records such as motor and orthophonic rehabilitation, and the average number of sessions prescribed.

Cost estimation

Costs were estimated using the "bottom-up micro-costing" approach [16]. To estimate the direct medical costs, we categorized disease costs into cost components: hospitalization (reanimation, intensive care, cardiology or neurology), medical consultation, biological tests, morphological explorations, drugs, and motor or orthophonic rehabilitation. Direct non-medical and indirect costs were not included.

The costs of ischemic stroke management were separated into three components: consultation and hospitalization, explorations, and treatment (drugs and motor and orthophonic rehabilitation). The pricing of all these components was obtained from the National Reference Pricing (TNR) established by the National Health Insurance agency (ANAM), as well as the General Nomenclature of Professional Acts (NGAP), the Nomenclature of Medical Biology Acts (NABM) and the conventions made under the supervision of ANAM between the organizations managing obligatory health insurance and different university hospital centers, the National Association of Private Clinics, public establishments of care and hospitalization, the Council of the Biologist Pharmacists, and the physicians and care Establishments of the liberal sector [17]. Costs were estimated from a social perspective. A one-year time horizon was adopted in the estimation, as recommended for prevalence-based studies.

Data analysis

The results are presented in three components: sociodemographic and clinical population characteristics and health service utilization, cost of ischemic stroke, and factors associated with a high cost of ischemic stroke. All costs were calculated in Moroccan dirhams (MAD) and then converted to US$ using an exchange rate of 1 USD = 9.595 MAD, as on December 31, 2019 [18]. The costs were adjusted by applying a discount rate of 3% and corrected by the annual Consumer Price Index (CPI). Costs were not discounted to present values if the time horizon did not exceed 12 months.

Categorical variables are presented in frequency counts and percentages, for quantitative variables in means ± standard deviations, and 95% confidence intervals (CIs) for all categories of cost. Costs were compared based on different factors (age, sex, complication, Rankin score, National Institutes of Health Stroke Scale, or NIHSS, score and length of hospitalization) using Student's t-test for comparison of two means or ANOVA for comparison of several means. The NIHSS is a tool used to objectively quantify the impairment caused by a stroke; it was designed for the National Institute of Neurological Disorders and Stroke (NINDS) and was published in 2001. It is composed of 11 items, each of which scores a specific deficit between a 0 and 4, with 0 representing normal function, while a higher score is indicative of some level of impairment. The Rankin Scale is used to assess the degree of disability in patients who have had a stroke. The score ranges from 0 to 6 with increasing severity, where 0 means no disability and 6 indicates death.

All tests were two-tailed, and the threshold of significance was p<0.05. Data entry and processing was done using Excel 2016 (Microsoft, Redmond, WA); for statistical analysis, SPSS Statistics, version 26 (IBM Corp., Armonk, NY) was used.

Ethical considerations

The study protocol was approved by the Ethics Committee of the Ibn Sina University Hospital, Rabat (reference 57/21).

## Results

Sociodemographic and clinical characteristics and health service utilization

A total of 267 individuals were included, with a female predominance (56.6%) and a female-to-male ratio of 1.3. The mean age was 66.93 ± 14.83 years, with 62.5% aged ≥65 years. A total of 98.5% of our patients had at least one brain CT scan done and 22.9% had at least three brain CT scans. In other morphological examinations, transthoracic ultrasound was the most performed procedure after CT with a frequency of 87.5%; the most performed biological tests were blood count, blood ionogram, renal function and C-reactive protein (CRP) tests with frequencies ranging from 91.1% to 98.1%.

The average length of hospital stay was 13.54 ± 9.72 days. The average Glasgow score on admission was 14.16 ± 2.06, with an initial NIHSS score of 12.07 ± 5.44; the majority of patients had a Rankin score ≥4 (58.8%). Complications were observed in 21.7% of patients, as indicated in Table 1.

**Table 1:** Clinical characteristics of stroke patients (n=267) NIHSS: National Institutes of Health Stroke Scale

	Women	Men	Total, mean (±SD)/n (%)
Age (years)	66.30	67.74	66.93 (14.83)
Age group			
<65	59	41	100 (37.5%)
≥65	92	75	167 (62.5%)
Sex	151 (56.6%)	116 (43.3%)	
Rankin score			
0	10	10	20 (7.5%)
1	15	9	24 (9%)
2	16	8	24 (9%)
3	23	19	42 (15.7%)
4	31	22	53 (19.9%)
5	47	43	90 (33.7%)
6	9	5	14 (5.2%)
Complication			
0	97	83	180 (78.3%)
1	30	20	50 (21.7%)
NIHSS score	12	12	12.07 (5.44)
Mode of exit			
Death	20	11	31 (11.9%)
Transfer	3	1	4 (1.5%)
At home	126	100	226 (86.6%)
Length of hospitalization	14	13	13.54 (9.72)

Cost of ischemic stroke management

The total average cost of ischemic stroke management per patient per year was estimated at $3674.32 ± 1340.81. The average cost of different components varied between $15.49 ± 15.10 for consultation and $1415.06 ± 1015.53 for hospitalization. The average cost of medical treatment was $766.39 ± 536.75 as summarized in Table 2.

**Table 2:** Cost of the different components of care

	Cost, mean (±SD)	95% CI
Cost of hospitalization	1415.06 (1015.53)	1292.65–1537.43
Cost of consultation	15.49 (15.10)	13.68–17.31
Cost of biological tests	220.32 (144.94)	202.86–237.79
Cost of radiological tests	412.87 (217.35)	386.68–439.06
Cost of medical treatment	766.39 (536.75)	701.71–831.07
Rehabilitation cost	844.19 (0)	844.19–844.19
Total cost	3674.32 (1340.81)	3512.76–3835.88

Concerning the univariate analysis, we found a statistically significant association between overall cost and age ($3934.24 for patients with age <65 years versus $3518.68 for those with age ≥65 years) (p=0.014), NIHSS score, with the total cost increasing with a higher NIHSS score at admission, B=580 (304; 857) (p≤0.001), and length of hospitalization, B=1180 (1107; 1252) (p≤0.001). On the other hand, no association was found with the infection (p=0.22) or with the other factors (sex, complication, Rankin score, etc.), with the significance level exceeding 0.2. Table 3 reflects these results. The multivariate analysis did not find a significant association, except for the length of hospitalization, which still remained significantly associated with the overall cost with p≤0.001.

**Table 3:** Comparison of costs based on different factors (univariate analysis) B is the correlation coefficient. W and M denote women and men, respectively.

		Total cost ($)	Significance level
Age (years)	<65	3934.24	0.014
	≥65	3518.68	
Sex	W	3649.52	0.73
	M	3706.59	
Complication	0	3640.96	0.26
	1	3923.30	
Rankin score	0	3636.15	0.68
	1	3543.74	
	2	3858.82	
	3	3791.23	
	4	3574.93	
	5	3757.79	
	6	3125.29	
NIHSS score	B = 580 (304; 857)		<0.001
Length of hospitalization	B = 1180 (1107; 1252)		<0.001

## Discussion

To our knowledge, this survey is the first to estimate ischemic stroke management costs in Morocco. The average direct cost of managing this condition was estimated to be $3674.32 (95% CI 3512.76-3835.88), which is 1.64 times higher than the average income per person reported by the High Commission for Planning (HCP) in 2019 ($2242) [19]. This finding highlights the financial burden placed on patients and their families when managing an ischemic stroke. It was also found that the cost of management increases with a higher NIHSS score at admission, indicating that more severe cases may lead to more complications, increased medication use and lengthier hospital stays, which can explain the high healthcare costs. Overall, these results provide valuable insights into the economic impact of managing ischemic stroke in Morocco and may help guide future healthcare policy decisions.

Our study aimed to assess the cost of stroke care in Morocco and compare it with previous studies conducted in different countries. We found that the cost of stroke care in our setting was almost similar to that reported in Lebanon ($3613) [20], Argentina ($3888) [21], and China ($3626) [22]. On the other hand, some studies, such as the one in Turkey by Asil et al. [23], in Pakistan by Khealani et al. [24], in Cameroon by Njankouo et al. [25], in Malaysia by Nor Azlin et al. [26], and in Senegal by Touré et al. [9], found too low costs: $1677, $1179, $1543, $807, and $416, respectively. In contrast, a study by Strilciuc et al. in Romania reported a higher cost of stroke care at $5226.82 [27]. The difference in the cost estimates among these studies may be due to the method used to estimate the cost of stroke care; for instance, Nor Azlin et al. adopted a top-down costing approach with a global vision that did not take into account the patient's share of spending (out-of-pocket) [26]. On the other hand, our study used a bottom-up approach based on a societal perspective that included details of spending from all contributors (state, insurance companies, and patients). Our study provides an estimate of the cost of stroke care in our setting that is comparable with previous studies in other countries. The differences in cost estimates among these studies highlight the importance of using an appropriate method to estimate the cost of stroke care, which considers all contributors and perspectives.

In terms of cost determinants, similar to our investigation, several studies have examined the relationship between the management costs of ischemic stroke and both the duration of hospitalization and the initial NIHSS score. These studies have highlighted the impact of these variables on healthcare expenditures, related to ischemic stroke, in countries such as Lebanon [20], Thailand [28], and Cameroon [24]. The length of hospitalization refers to the duration of a patient's stay in the hospital, while the NIHSS score measures stroke severity. These factors have been recognized as significant contributors to healthcare costs due to their influence on the resources and services required for stroke treatment.

Unlike other surveys, in our study, infection was not significantly associated with cost [29]. One possible explanation for this finding is that a large proportion of infected patients (44.9%) actually died during hospitalization, which may have resulted in less expenses compared to patients who survived longer and required more intensive care.

Overall, these studies highlight the fact that the cost of stroke care is a complex and multifactorial issue that can be affected by various factors. Identifying these factors and understanding their impact on cost can help healthcare providers to optimize treatment and minimize costs without compromising patient outcomes. Several strategies can be adopted to minimize costs, in particular, the adoption of teleneurology networks to improve access to thrombolysis for acute ischemic stroke, improving efficiency of emergency care for acute ischemic stroke, and provide alternatives to hospital care [30].

Some method limitations need to be discussed. First, we restricted our study to the public health sector (university hospital). The limitation of our research to this specific sector may have resulted in an underestimation of the true cost of managing ischemic strokes. Secondly, the data were collected retrospectively from medical records. Face-to-face questionnaires would allow for the collection of more data.

Overall, despite these limitations, this scientific study provides valuable insights into the cost of managing ischemic strokes in the public health sector. Future research could build upon this study to further improve our understanding of the costs and challenges of managing ischemic strokes in healthcare settings. Furthermore, the study setting was the neurology department of the Hassan II University Hospital in Fez. It is a reference center for stroke management and handles different regions reflecting the ethnic and sociodemographic characteristics of the whole Moroccan territory. Moreover, we adopted a "bottom-up" approach with a societal perspective, which favors the direct assessment of unit costs for each resource used in the treatment process of a particular type of patient (diagnosis, consultation, hospitalization, complementary tests, etc.). This allowed us to better reflect reality and to have greater precision than other approaches.

## Conclusions

Stroke incurs considerable economic costs in Morocco. Because the Moroccan population is aging rapidly, the societal burden of care may increase significantly in the future. Efforts to control service price and strength of healthcare services, such as novel approaches to incentivize prevention and high-value care, may be needed to contain anticipated increases in spendings. Cost-effectiveness analyses and budget impact analyses in local health systems would be useful for specific policy proposals.
